# Next Generation Sequencing Reveals Regulation of Distinct *Aedes* microRNAs during Chikungunya Virus Development

**DOI:** 10.1371/journal.pntd.0002616

**Published:** 2014-01-09

**Authors:** Jatin Shrinet, Shanu Jain, Jaspreet Jain, Raj K. Bhatnagar, Sujatha Sunil

**Affiliations:** International Centre for Genetic Engineering and Biotechnology, New Delhi, India; National Institute of Allergy and Infectious Diseases, United States of America

## Abstract

**Background:**

Application of genomics and Next Generation sequencing has led to the identification of new class of cellular functional molecules, namely, small RNAs. Of the several classes of ncRNAs (non-coding RNA), microRNAs have been demonstrated to exert determinative influence on various cellular processes. It is becoming abundantly clear that host/vector/pathogen encoded microRNAs impact eventual pathogenesis. In this context, the participation of vector based microRNAs in disease transmission and pathogen development is being investigated intensively. A few studies have highlighted the role of vector encoded microRNAs in pathogen infection. We conducted this study to evaluate the role of host miRNAs upon CHIKV (Chikungunya Virus) infection in an important vector, *Aedes albopictus*.

**Findings:**

We identified 88 and 79 known miRNAs in uninfected and CHIKV infected *Ae. albopictus* Singh's cell line respectively. We further identified nine novel miRNAs in *Ae. albopictus*. Comparison of the two libraries revealed differential expression of 77 common miRNAs between them. CHIKV infection specifically altered the miRNA profile of a specific set of eight miRNAs. Putative targets of these regulated miRNAs were identified and classified into their pathways.

**Conclusions:**

In our study we have identified and described the profiles of various miRNAs upon CHIKV infection in *Ae. albopictus*. This investigation provides an insight about cellular modification by miRNAs during CHIKV infection and the results provide leads for identifying potential candidates for vector based antiviral strategies.

## Author Summary

Small, non-coding, RNAs such as microRNAs (miRNA) of length 18–24 nucleotide are reported in wide range of organisms and known to play a determinative role in regulation of various cellular processes. They have also reported to have regulatory roles during pathogen infection, maturation and transmission. Chikungunya virus is an important alphavirus transmitted through two important vectors, *Aedes aegypti* and *Ae. albopictus*. While *Ae. aegypti* has been the preferred vector for transmission of this virus, recently, *Ae. albopictus* has gained notoriety for disease transmission. Using Illumina platform, we sequenced the small RNA population an *Aedes albopictus* cell line infected with chikungunya virus. We studied the expression modulation of miRNAs upon CHIKV infection and found distinct set of miRNAs regulated in Aedes upon CHIKV infection. We further predicted the targets of these miRNAs and performed pathway analysis of these targets. The cellular functional targets of some of these miRNAs overlapped suggesting aggressive participation of the targeted pathways in establishing CHIKV infection.

## Introduction

Vector borne diseases like malaria, dengue, chikungunya are a major burden to the economy of countries in the tropical and sub-tropical regions. The situation in Asian countries like India are especially alarming with recent studies predicting India to have the maximum global burden of dengue [1] and encompassing nearly 46% of global population at risk for vivax malaria [2]. Currently, no vaccines are available for any of the above mentioned infections and vector control with intense public awareness is the only effective measure for disease control. Transmission blocking approaches provide hope for disease control in laboratory conditions [3] which clearly emphasizes the importance of understanding vector-pathogen dynamics for effective control strategies.

Insects play an important role in disease transmission in plants, animals and humans. Mosquitoes are especially significant in disease transmission in humans. Fundamental biological process of blood feeding in female mosquitoes for egg development is utilized by pathogens either for completing their life cycle or amplifying within the mosquito before infecting the mammalian hosts. Until now, mechanisms involved in disease transmission were obscure. With the recent onslaught of several arboviral epidemics and increase of vector transmitted diseases, several studies, both genetic and non-genetic, have established the molecular mechanisms that may be involved in the host seeking behavior of mosquitoes [4], [5], their response to pathogens [6], [7] and those factors of pathogen development within the vector affecting transmission [8], [9].

RNA interference is the most important phenomena used by insects as the first line of defense against microbial invaders and has been extensively studied in the recent times [10]–[13]. Infection of mosquitoes by virus results in generation of different kind of small RNA populations of viral origin, namely 22 nt siRNA population [14], longer nt piRNA population [15], [16] as well as modulation of host miRNAs [17]–[19]. MicroRNAs are a class of small (19–24 nt in length), non-coding, single stranded RNAs known to post-transcriptionally regulate gene expression by binding to 3′ untranslated regions (3′UTRs) of the target mRNAs, resulting in translational repression/cleavage of the cognate mRNAs. First identified in *Caenorhabditis elegans*, miRNAs have since been identified in a variety of organisms including plants, animals, insects and viruses [20]. Their function primarily is regulation of gene expression and play crucial roles in cell development, proliferation, differentiation, host defense, fat metabolism and apoptosis [21].

We undertook the present study to identify mosquito host miRNAs that may be impacted during chikungunya infection. For this purpose, we performed deep sequencing of small RNA population in *Aedes albopictus* Singh's cell line upon chikungunya virus infection. We identified several Aedes known and novel miRNAs and profiled their expression during CHIKV infection. In a separate study we had identified microRNAs in *Anopheles stephensi* mosquitoes and profiled these miRNAs upon *Plasmodium* infection [22]. During the course of analysis, we recognized there were miRNAs common between *Aedes* and *Anopheles*; however, there was discrete regulation of some of the common miRNAs upon infection by CHIKV and *Plasmodium*. In this report, we describe *Ae. Albopictus* miRNAs and their regulation upon CHIKV infection. Furthermore, we highlight the repertoire of common and distinct host miRNAs regulated during *Plasmodium* and CHIKV infections in their respective hosts. We also have predicted the targets of some of these miRNAs in a bid to understand the role these miRNAs may be playing during pathogen infection in mosquitoes.

## Methods

### Cell culture and virus infection

*Ae. albopictus* cell line Singh's line (ATCC-CCL-126) was maintained at 28°C in DMEM media supplemented with 10% FBS and antibiotics. The cells were infected with MOI = 10 of the wild type chikungunya virus (Accession no. JF950631.1) isolated from India during an outbreak in 2010 [23]. Cells were infected in triplicates and harvested 24 hours post infection and processed further for small RNA sequencing.

### Small RNA sequencing

Illumina Truseq small RNA libraries for two samples, namely, *Ae. Albopictus* Singh's cell line uninfected (SL) and *Ae. albopictus* Singh's cell line infected with CHIKV (iSL) were made using manufacturer's instructions (Illumina Inc). Briefly, 1 ug of total RNA was ligated with 3′and 5′adaptors in sequential steps and reverse transcribed using RT primers. Following amplification of the adaptor enriched fragments, population of small RNA within length 140–160 bps were eluted from 6% TBE PAGE gel. Eluted products were precipitated using sodium acetate and ethanol and dissolved in RNAse free water. The samples were quantified using the Total RNA Nano chip in Bioanalyzer. Deep sequencing was performed using Illumina Genome Analyzer II.

### Data analysis pipeline

#### Identification of known microRNAs

Data was analyzed using an in-house pipeline developed for this purpose (Figure S1). Mature miRNA sequences and pre-miRNA sequences of all insect species available including *Aedes aegypti* (Aae), *Anopheles gambiae* (Aga), *Acyrthosiphon pisum* (Api), *Apis mellifera* (Ame), *Bombyx mori* (Bmo), *Culex quinquefasciatus* (Cqu), *Drosophila melanogaster* (Dme) and *Tribolium castaneum*(Tca) were downloaded from miRBase database V.19 [24]. All the sequences were combined for the analysis and 100% similar sequences were pooled together using CD-HIT web tool [25] to reduce data redundancy. Other non-coding RNAs (ncRNA) were downloaded from ncRNA database [26]. Sequences representing coding region of *Aedes aegypti, Anopheles stephensi* and *An. gambiae* genomes were also downloaded from Vectorbase [27] using BioMart tool. The tools Bowtie, RNAfold and RNAplot used in this study were downloaded and incorporated in our in-house PERL based pipeline developed for small RNA analysis. All the downloaded sequences were indexed using Bowtie [28] and used for the analysis. Analysis for all libraries was performed separately. Reads derived from deep sequencing were trimmed and filtered to fetch sequences having length> = 18 bases. Hundred percent matched sequences were pooled together to generate fasta files (expression file) having the unique number along with read counts for each of the unique sequence as an ID, for further analysis. The filtered reads (length> = 18) were aligned against the mature miRNA sequences using Bowtie allowing one mismatches and taking other parameters as default.

#### Identification of novel miRNA

For identification of novel miRNAs, unmatched sequences after the known miRNA prediction were mapped to pre-miRNA sequences keeping the same parameters as used for known miRNA prediction. For prediction of novel miRNAs, the pre-miRNA unmatched reads were mapped to ncRNA sequences to filter out any other non-coding RNAs from the libraries. The unmatched sequences after this step were then matched to coding region of *Ae. aegypti, An. gambie and An. stephensi* to filter out the sequences falling in coding regions. The final set of unmatched sequences were then matched to respective genomes and matched sequences were subjected to novel miRNA prediction pipeline which uses RNAfold and RNAplot [29] for secondary structure prediction of pre-miRNA and mapping of small RNA into the pre-miRNA. Those sequences matching to the genome, 75 nt flanking region from both sides of the small RNA sequences were fetched as precursor sequences. These pre-sequences were then folded and energies were calculated by RNAfold and RNAplot. Novel miRNA sequences were then filtered on the basis three main criteria: the hairpin loop structure of the fetched precursor sequence, the presence of the small RNA sequence in any of the arm of precursor sequence, energy < = −20 KJ/mol.

#### Relative abundance and expression profiling of microRNAs from small RNA sequencing libraries

miRNA abundance in both libraries were calculated after normalizing the reads. The samples were normalized by calculating tag per million of total RNA reads (TPM) and was used for comparing relative abundance of specific microRNAs within each data set. For the purpose of identification of known miRNAs from different organisms, our pipeline was designed in a manner to treat reads with even single nucleotide variation and also reads with different lengths as a distinct small RNA. However, for profiling the relative abundance of miRNAs, we combined all read counts with same miRNA names so as to reduce redundancy in data and to increase accuracy of the reads count of the miRNAs. The miRNAs in iSL (Singh's cell line infected with CHIKV) were further profiled against the SL (Singh's cell line uninfected) to identify the differentially expressed miRNAs upon CHIKV infection.

#### Expression profiling of miRNA by real time PCR

From the list of miRNAs, two miRNAs namely miR-281-5p and miR-75p were selected at random. Real time PCR analysis was performed using ncode real time PCR kit (cat. MIRCQ 100). Real time PCR was set up in triplicates for miRNAs of uninfected and 24 hours CHIKV infected cells as per the manufacturer's instructions. 5.8 s rRNA was used as an endogenous control for miRNA expression profiling. Expression levels were then calculated against SL as a calibrator using 2^−ΔΔC^_T_ method.

#### Comparison of miRNA expression profiles in different vectors upon pathogen infection

miRNAs identified in the present study were compared with miRNAs identified in a recent study [22] to understand the behavior of these miRNAs in different mosquitoes infected by pathogens specific to them. miRNAs that were common were analyzed to understand mosquito/pathogen specific miRNA regulation.

#### Statistical analysis

Statistical tests for identifying significant differentially expressed miRNAs were performed using edgeR module with few modifications in the script. The pvalue cutoff was performed on the data with the significance threshold selected as 0.05.

#### Target prediction of microRNAs and pathway analysis

For identifying putative targets of differentially up regulated miRNAs, 3′ UTR sequences of *Ae. aegypti* and *An. gambiae* were downloaded from VectorBase and were subjected to target prediction using RNA hybrid tool [30]. The targets were filtered on the basis of complementarity of the miRNA with the targets and energy of the miRNA:target duplex< = −20 Kcal/mol. The targets of selected down-regulated and up-regulated miRNAs were further subjected to KOBAS analysis for the identification of significant pathways, selecting the species as *Ae. aegypti* and *An. gambiae* and KEGG as pathway database keeping the threshold p value ≤0.05 [31]. The vectorbase ID for the targets of significant pathways were then used to generate the miRNA:mRNA interaction network. Cytoscape was used for visualizing the networks [32].

## Results

### Characterization of Aedes miRNAs in *Ae. albopictus* cell line

To evaluate the impact of CHIKV infection on *Ae. albopictus* miRNAs, we carried out high throughput small RNA sequencing using Illumina platform on small RNA libraries obtained from CHIKV infected *Ae. albopictus* cell line Singh's line and compared it with uninfected cell line. Details of small RNA sequencing data generation of the two libraries and subsequent analyses are detailed in **Table 1**. In the libraries, there was an overall reduced number of reads in iSL as opposed to SL which could be due to the impact of infection. There was a bimodal pattern of reads, one distinct peak at 22 nt and another peak at 27–30 nt in both libraries. A total of 79.19% reads of SL, 81.94% reads of iSL were mappable and these reads were mapped against *Ae. aegypti* genome. Analysis pipeline for identification of known microRNAs included zero and one mismatch to accommodate the sequence differences between *Ae. albopictus* and *Ae. aegypti*. The remaining raw reads were then mapped against chikungunya genome to discover CHIKV encoded small RNA population. We found a total of 18589 reads (> = 16 nt) mapping against chikungunya genome which was too low. We suspect that the overall lesser number of reads in the infected library to be a reason for this reduced number of virus derived small RNA population.

**Table 1:** Details of small RNA sequencing information and subsequent data analysis.

Sample name	Raw reads	Reads after adaptor trimming	ncRNA reads					Reads mapped to known microRNA	Total miRNA identified	CHIKV mapped reads
			tRNA	rRNA	snRNA, splicing	snRNA; snoRNA; CD-box;	HACA-box;			
**SL**	8647241	6848379	32270	16010	4677	4280	874	1646718	89	0
**iSL**	4618659	3784535	24663	9294	1869	2406	476	758926	80	18589

Mapping of reads from both SL and iSL against known mature miRNA library resulted in identification of 88 and 79 miRNAs of SL and iSL respectively (**Table 2**). Among the identified miRNAs, 36 were conserved in three species of mosquitoes (*Aedes*, *Anopheles* and *Culex*) and 12 were conserved in all the eight species we used for our analysis.

**Table 2:** Details of total miRNAs identified (0 mismatch) in *Aedes albopictus*.

S. No.	Sequence	miRNA	Singh's cell line	Infected Singh's cell line
1	TGAGATCATTTTGAAAGCTGATT	bantam-3p	11771.15	7066.99
2	CCGGTTTTCATTTTCGATCTGAC	bantam-5p	17.81	23.17
3	TGAGGTAGTTGGTTGTATAGT	let-7	2786.44	1912.68
4	TGGAATGTAAAGAAGTATGG	miR-1	0.12	0
5	AACCCGTAGATCCGAACTTGTG	miR-100	4167.34	5236.15
6	ATATTGTCCTGTCACAGCAGT	miR-1000	3.82	0.87
7	TACCCTGTAGAACCGAATTTGT	miR-10-5p	4.51	2.6
8	CATCACAGTCAGAGTTCTTGCT	miR-11-3p	6812.69	4749.86
9	TGAGATTCTACTTCTCCGACT	miR-1175-3p	0.58	0
10	ACAAGTTTTGATCTCCGGTAT	miR-125-3p	38.86	64.3
11	TCCCTGAGACCCTAACTTGTGA	miR-125-5p	270.61	327.58
12	TGAGTATTACATCAGGTACTGGT	miR-12-5p	383.82	295.76
13	TTGGTCCCCTTCAACCAGCTGT	miR-133-3p	1.5	0.65
14	TATCACAGCCATTTTGACGAGTT	miR-13-3p	539.59	332.78
15	TCGTAAAAATGGTTGTGCTGT	miR-13-5p	20.47	16.02
16	TATTGCTTGAGAATACACGTAG	miR-137	0.35	0
17	TCAGTCTTTTTCTCTCTCCT	miR-14	7725.12	5398.97
18	TGGACGGAGAACTGATAAGGGC	miR-184-3p	81027.58	83651.55
19	TGAAATCTTTGATTAGGTCTGG	miR-1890	24.52	16.45
20	TGAGGAGTTAATTTGCGTGTT	miR-1891	0	0.22
21	CCCAGGAATCAAACATATTATT	miR-190-3p	0.46	0.22
22	AGATATGTTTGATATTCTTGGTTG	miR-190-5p	219.38	78.59
23	TACTGGCCTACTAAGTCCCAA	miR-193	0.12	0
24	CTTGTGCGTGTGACAACGGCT	miR-210-3p	0.93	0.87
25	AGCTGCTGACCACTGCACAAGA	miR-210-5p	0.12	0
26	TGATTGTCCAAACGCAATTCTTG	miR-219	0.23	0
27	CTGCTGCCCAAGTGCTTAT	miR-252-3p	12.26	5.63
28	CTAAGTACTAGTGCCGCAGGA	miR-252-5p	1719.74	1768.7
29	AATGGCACTGGAAGAATTCAC	miR-263a-5p	8.21	4.55
30	TCAGGTACCTGAAGTAGCGCGC	miR-275-3p	2392.56	3182.31
31	TAGGAACTTCATACCGTGCTCT	miR-276-3p	4000.47	4445.45
32	TGGTAACTCCACCACCGTTGGC	miR-2765	191.16	161.95
33	AGCGAGGTATAGAGTTCCTACG	miR-276-5p	3.35	3.25
34	TAAATGCACTATCTGGTACGAC	miR-277-3p	2673.22	2318.64
35	ATCCGGCTCGAAGGACCA	miR-2779	13.18	14.72
36	TCGGTGGGACTTTCGTCCGTT	miR-278-3p	60.83	36.37
37	TGACTAGATCCACACTCATT	miR-279	338.95	281.03
38	GTAGGCCGGCGGAAACTACTTGC	miR-2796	0.12	0
39	TGTCATGGAATTGCTCTCTTTA	miR-281-3p	20.82	15.59
40	AAGAGAGCTGTCCGTCGACAGT	miR-281-5p	4829.63	5092.82
41	CAATATCAGCTGGTAATTCTG	miR-283	1.73	5.85
42	TAGCACCATTCGAAATCAGTAC	miR-285	0.46	0.22
43	TGACTAGACCGAACACTCGCGT	miR-286a	5.32	6.06
44	TGACTAGACCGAACACTCGTGTCC	miR-286b	0	12.34
45	GTCGACAGGGAGATAAATCACT	miR-2940-3p	32669.5	16188.68
46	TATCACAGCAGTAGTTACCT	miR-2944a-3p	0.12	0
47	TATCACAGCAGTAGTTACC	miR-2944b-3p	4.39	4.76
48	GAAGGAACTCCCGGTGTGATATT	miR-2944b-5p	13.65	15.37
49	TGACTAGAGGCAGACTCGTTT	miR-2945-3p	279.05	166.07
50	AGCTCAGCACGCAGGGGC	miR-2951-5p	0.35	0.43
51	TATCACAGCCAGCTTTGAAGA	miR-2a-3p	1043.34	911.52
52	TATCACAGCCAGCTTTGATGAGCT	miR-2b	925.73	348.15
53	TATCACAGCCAGCTTTGA	miR-2c-3p	11.56	2.38
54	CGGCACATGTTGGAGTACACTT	miR-305-3p	111.25	319.79
55	ATTGTACTTCATCAGGTGCTCTGG	miR-305-5p	689.82	509.24
56	GAGAGCACCTCGGTATCTAAGC	miR-306-3p	0.35	0.87
57	TCACAACCTCCTTGAGTGAGC	miR-307	1.62	2.17
58	AATCACAGGAGTATACTGT	miR-308-3p	23.71	21.22
59	TCACTGGGCAAAGTTTGTCGC	miR-309a	43.37	32.69
60	TGGCAAGATGTTGGCATAGCT	miR-31	0.58	0.22
61	TTTTGATTGTTGCTCAGAAAGCC	miR-315	1.04	0.22
62	GTGCATTGTAGTTGCATTGCA	miR-33-5p	35.97	15.81
63	TGGCAGTGTGGTTAGCTGGTTGT	miR-34-5p	89.05	59.76
64	TTTGTTCGTTTGGCTCGAGTTA	miR-375	3.58	1.73
65	TCTCACTACCTTGTCTTTCA	miR-71-3p	745.9	596.28
66	AGAAAGACATGGGTAGTGAGAT	miR-71-5p	20.47	20.14
67	TGGAAGACTAGTGATTTTGTTGT	miR-7-5p	15.03	6.71
68	ATAAAGCTAGATTACCAAAGC	miR-79-3p	16.07	14.51
69	CTTTGGCGCTTTAGCTGTATGA	miR-79-5p	1.04	1.52
70	TAATACTGTCAGGTAAAGATGTC	miR-8-3p	2808.76	2397.23
71	CATCTTACCGGGCAGCATTAGA	miR-8-5p	281.01	457.28
72	TTTAGAATTCCTACGCTTTACC	miR-927a	0.46	1.73
73	AAATTGACTCTAGTAGGGAGT	miR-929-5p	0.46	0.87
74	TATTGCACTTGTCCCGGCCT	miR-92a-3p	14.46	22.3
75	AATTGCACTTGTCCCGGCCTGC	miR-92b-3p	128.48	95.48
76	TCAATTCCGTAGTGCATTGCAGT	miR-932-5p	0.12	0
77	TGAAACCGTCCAAAACTGAGGC	miR-957	1.73	1.3
78	TAAGCGTATAGCTTTTCCCAT	miR-965	1.39	0.87
79	TCATAAGACACACGCGGCTAT	miR-970	558.56	514.44
80	TAGCTGCCTAGTGAAGGGCT	miR-980-3p	333.86	282.55
81	CCCCTTGTTGCAAACCTCACGC	miR-988-3p	58.17	71.88
82	GTGTGCTTTGTGACAATGAGA	miR-988-5p	0.46	0
83	TGTGATGTGACGTAGTGGTAC	miR-989	0.46	0.22
84	TGACTAGATTACATGCTCGT	miR-996	263.9	205.25
85	TAGCACCATGAGATTCAGCTC	miR-998-3p	47.88	58.89
86	TGTTAACTGTAAGACTGTGTCT	miR-999	349.13	249.21
87	TCTTTGGTTATCTAGCTGTAT	miR-9a	0.23	0.87
88	TAAAGCTTTAGTACCAGAGGTC	miR-9c-3p	100.73	119.08
89	TCTTTGGTATTCTAGCTGTAGA	miR-9c-5p	133.11	78.16
90	TTACGTATACTGAAGGTAT	miR-iab-8-5p	0.12	0

Furthermore, a total of nine novel miRNAs were identified in both the libraries.Out of these miRNAs, novel-miR-2 was found to be having miR*.The structures of these novel miRNAs are shown in **Figure S2**.

Mapping of known miRNAs was performed using the genome of *Ae. aegypti* as the genome of *Aedes albopictus* is not available. To accommodate the difference in the genome sequences, we extended the analysis to include miRNA reads having upto 2 mismatches with *Ae. Aegypti* genome. Our analysis with one mismatch revealed additionally29 miRNAs in the libraries, namely, miR-11-5p, miR-12-3p, miR-184-5p, miR-1889-3p, miR-1889-5p, miR-275-5p, miR-277-5p, miR-278-5p, miR-286-3p, miR-2940-5p, miR-2941, miR-2945-5p, miR-296, miR-2951-3p, miR-2a-5p, miR-306-5p, miR-308-5p, miR-309b-3p, miR-316, miR-317-3p, miR-317-5p, miR-34-3p, miR-745-3p, miR-87, miR-92a-5p, miR-92b-5p, miR-980-5p, miR-998-5p and miR-9b. The identification of 29 new miRNA after allowing 1 mismatch also indicates the differences in *Aedes aegypti* and *Aedes albopictus* genomes (**Table 3**).

**Table 3:** Details of additional 29 miRNAs identified after 1 mismatch data analysis in *Aedes albopictus*.

miRNA	Sequence	TPM (SL)	TPM (iSL)
miR-11-5p	CAAGAACTCCGGCTGTGACC	9.945368702	15.80545349
miR-12-3p	CAGTACTTATGTTATGCTCTCT	94.94357796	94.18318174
miR-184-5p	CCTTATCATTCTTTCGCCCCG	9.251505769	8.010983274
miR-1889-3p	CACGTTACAGATTGGGGTTTCC	39.2032557	44.60169066
miR-1889-5p	TAATCTCAAATTGTAACAGTGG	296.8576914	227.9882537
miR-275-5p	CGCGCTAAGCAGGAACCGAGAC	7.169916971	22.08433227
miR-277-5p	CGTGTCAGAAGTGCATTTACA	6.938629327	10.60914001
miR-278-5p	ACGGACGATAGTCTTCAGCGGCC	28.2170926	37.67327269
miR-286-3p	TGACTAGACCGAACACTCGTGT	405.4472403	466.5856474
miR-2940-5p	TGGTTTATCTTATCTGTCGAGGC	5736.280508	4856.820995
miR-2941	TAGTACGGCTAGAACTCCACGG	47.41396707	57.59247435
miR-2945-5p	AGCGGGTCCGTTTCTAGTGTCATG	7.63249226	8.444009396
miR-2946	TAGTACGGAAAAGATATGGGGA	7.63249226	6.495391843
miR-2951-3p	TCTGCCCGGTTCCGTGTACTG	11.56438221	14.07334899
miR-2a-5p	ACTCTCAAAGTGGCTGTGAAA	11.68002603	18.62012328
miR-306-5p	TCAGGTACTCAGTGACTCTCA	5063.696039	4191.69287
miR-308-5p	CGCGGTATATTCTTGTGGCTT	21.04717563	48.71543883
miR-309b-3p	TCACTGGGCATAGTTTGTCGC	0.809506755	0.433026123
miR-316	TGTCTTTTTCCGCTTACTGCCT	0.115643822	0.216513061
miR-317-3p	TGAACACAGCTGGTGGTATCT	1835.036169	1918.089212
miR-317-5p	CGGGATACACCCTGTGCTCGCT	74.12768998	153.5077606
miR-34-3p	CAACCACTATCCGCCCTGCCGCC	43.59772094	76.86213682
miR-745-3p	TAGCTGCCTAGCGAAGGGCA	0.346931466	0.216513061
miR-87	GTGAGCAAATTTTCAGGTGTGT	37.93117365	18.40361022
miR-92a-5p	CGGTACGGACAGGGGCAACATT	0.346931466	0.649539184
miR-92b-5p	AGGTCGTGACTTGTGCCTGTTTG	0.115643822	0
miR-980-5p	CGGCCGTTCATTGGGTCATCTAGC	7.516848438	20.56874084
miR-998-5p	ACTGAACTCTCGTGGGTCTGCA	1.156438221	3.03118286
miR-9b	TCTTTGGTGATTTTAGCTGTATG	40.59098156	18.83663635

### Distribution and abundance of *Aedes* miRNAs

Further to identification of *Aedes* miRNAs, we analyzed the distribution and abundance of these miRNAs within the libraries. Among 90 miRNAs identified in this study, majority of miRNAs were found in both the libraries (n = 77). Eleven miRNAs were found only in uninfected SL (miR-1175-3p,miR-988-5p,miR-137,miR-219,miR-1,miR-193,miR-210-5p,miR-2796,miR-2944a-3p,miR-932-5p,miR-iab-8-5p), while two miRNAs (miR-286b and miR-1891) were found to be unique to iSL. When we performed the analysis with 1 mismatch, we were able to identify miR-286b reads in SL; however, miR-1891 was absent in uninfected library in 1 mismatch data (**Table 3**). With respect to novel miRNAs, all nine novel miRNA were found to be present in both SL and iSL libraries. Out of these miRNAs, two miRNAs, novel-miR-4 and novel-miR-9 showed significant up-regulation in iSL and 5 miRNAs, novel-miR1-5p, novel-miR-2-3p, novel-miR-5, novel-miR-7 and novel-miR-8 showed significant down-regulation in iSL library (**Table 4**).

**Table 4:** Novel miRNAs identified in *Aedes albopictus*.

miRNA	Sequence	SL	iSL
novel-miR-1-5p	ATTAGAATGTGGAATCTGTTTT	0.35	0.12
novel-miR-2-5p	TATTCGCATAACAGTCCCATG	3.3536	3.0311
novel-miR-2-3p	CATGGGACTGTTATGCGAATA	7.7481	6.7119
novel-miR-3	AATTCGTGTACTTGGGCTC	0.6938	0.433
novel-miR-4	AGGCTTGTATGAATGGTTGAATGA	0.8095	1.299
novel-miR-5	CGTGATATTATCACTACGGA	1.7346	0.6495
novel-miR-6	TTCTGACGAAGCCCCTTGGTAC	1.8503	1.5155
novel-miR-7	TTTCGGATATGTTTTAGAAATTCGTT	192.893	139.8674
novel-miR-8	TGTATCGTATGTCACACTCGAGGCCCT	1.1564	0
novel-miR-9	TATTTCGTCCTTCAAACCCGCCT	23.1287	45.2512

Using the read counts, we further calculated the abundance of the miRNAs in the libraries. Using TPM values, the miRNAs were categorized into (i) significantly abundant (SA) with TPM greater than equal to 1000, (ii) moderately Abundant (MA) with TPM 100–999, (iii) abundant (A) with values ranging from 10–99 and (iv) rare (R) with values less than 10. In case of SL, 53 were found to be abundant, while in iSL, 51 were found to be abundant. Several miRNAs in iSL was rare (n = 29) while in SL, the number of miRNAs with counts less than 10 were 36 (**Table 2**). miR-184-3p was found to be the most abundant in SL as well as in iSL.

### Regulation of Aedes miRNAs upon CHIKV infection

**Figure 1:**
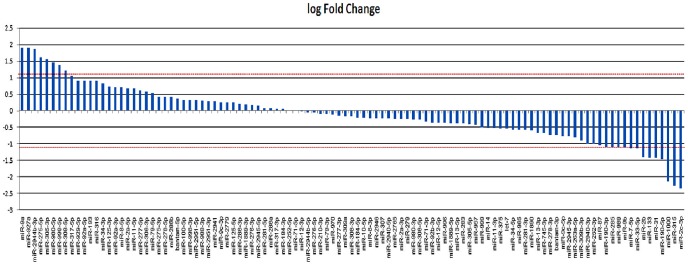
The figure represents details of LogFold change ratios of miRNAs identified in SL and iSL libraries.

Log fold change of miRNAs used in our study is depicted in **Fig. 1**. Statistical analysis performed using the edgeR package revealed 41 miRNAs to be significant differentially expressed in *Aedes* (p value<0.05). It is noteworthy that most of the miRNAs were down-regulated upon CHIKV infection. Based on p-value and log fold change, we identified four miRNAs (miR-100, miR-283, miR-305-3p and miR-927) to be significantly over-expressed and four (miR-1000, miR-2b, miR-2c-3p and miR-190-5p) to be under-expressed.

**Figure 2:**
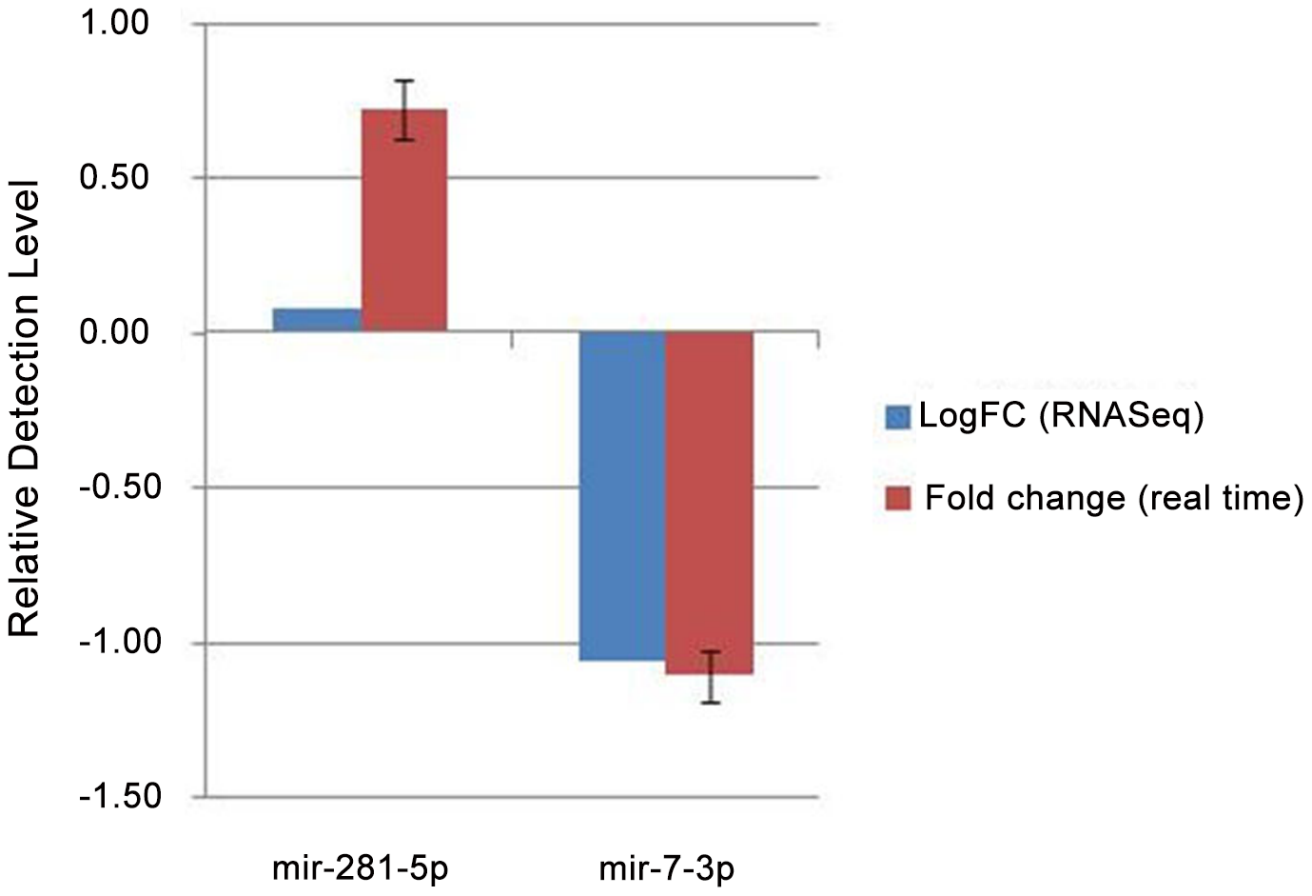
Real Time PCR data analysis of miR-281-5p and miR-7-3p. Fold change value obtained from RNA-Seq analysis are also represented in the figure.

To further validate the expression of these miRNAs, real- time PCR was performed on SL and iSL with miR-281-5p and miR-7 (**Fig. 2**). The miRNA expression profiles were found to be comparable to that derived from small RNA sequencing. The amplified products were cloned and sequenced.

### Target prediction and network analysis

**Figure 3:**
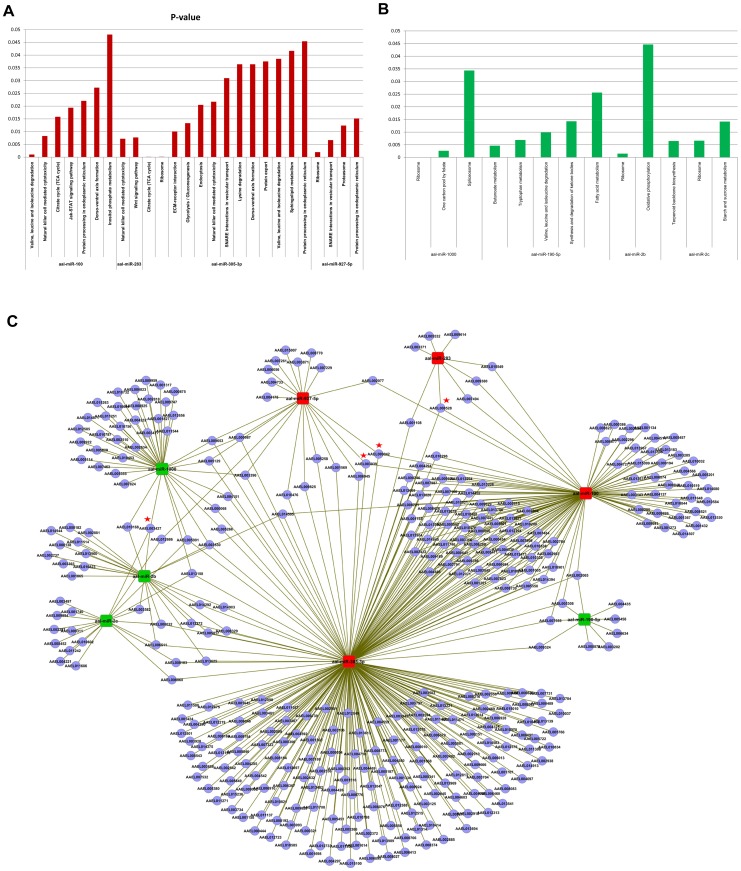
miRNA-target KOBAS analysis and interaction network of significantly up-regulated and down-regulated miRNAs of *Ae. aegypti*. (**A**) Graph represents significant pathways of up-regulated miRNAs after KOBAS analysis. (**B**) Graph represents significant pathways of down-regulated miRNAs after KOBAS analysis. (**C**) The targets common among the miRNAs are represented by red color star. Up-regulated miRNAs are shown by red colour rectangle and green color rectangles represent down-regulated miRNAs. Names of all miRNAs and targets are shown in figures.

Further to miRNA profiling, efforts were taken to identify putative targets of all the miRNAs that were regulated in *Aedes* upon CHIKV infection. Four significantly over-expressed miRNAs (miR-100, miR-283, miR-305-3p and miR-927) and 4 under-expressed miRNAs (miR-1000,miR-2b,miR-2c-3p and miR-190-5p) were analyzed for their putative targets. For the over-expressed miRNAs, 375 targets were identified for miR-100, 469 for miR-305-5p, 108 for miR-927, 186 for miR-283 and 395 targets for aal-miR-2944b-5p. For down-regulated miRNAs, 460 targets were identified for miR-1000, 226 for miR-2b, 263 for miR-2c-3p and 193 putative targets for miR-190-5p. These targets were further analyzed and clustered using KOBAS and the targets of the significantly represented pathways (p value<0.05) studied (**Figure 3a**). KOBAS analysis for up-regulated miRNAs revealed that the four up-regulated miRNAs targets the mRNAs playing role in seventeen different pathways. Out of all these pathways, Natural killer cell mediated cytotoxicity and Protein processing in endoplasmic reticulum pathways were common among miR-100, miR-283 and miR-305-3p. Citrate cycle (TCA cycle),dorso-ventral axis formation and valine, leucine and isoleucine degradation were found to be common between miR-100 and miR-305-3p and SNARE interactions in vesicular transport was commonly targeted by miR-927 and miR-305-3p. miR-305-3p was found to be mainly targeting the pathways essential for viral entry such as,ECM-receptor interaction,endocytosis,SNARE interactions in vesicular transport. Likewise in the case of down-regulated miRNAs, ribosome pathway was found to be common among miR-1000, miR- 2b and miR-2c.

The interaction of these targets was studied and the miRNA:mRNA interaction network is shown in **Fig. 3b**. Several transcripts were targeted by two or more up-regulated and down-regulated miRNAs. However, three transcripts, AAEL008528, AAEL013939 and AAEL009042, were found to be common targets among the up-regulated miRNAs and transcript, AAEL003427 was found to be a common target among the down-regulated miRNAs. Detailed investigations are important to understand the role of these mRNAs during CHIKV development in *Aedes*.

### miRNA regulation is specific to vector and pathogen

**Figure 4:**
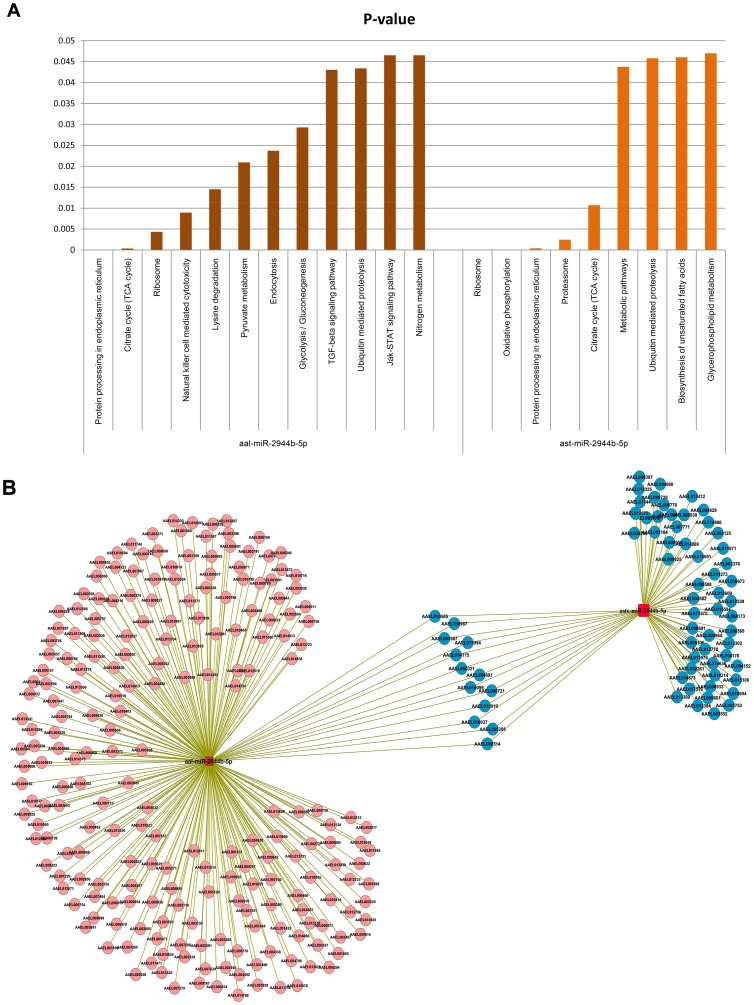
miRNA-targets KOBAS analysis and interaction network of miR-2944b-5p of *Ae. aegypti* and *An. stephensi*. (**A**) Graph represents significant pathways of aal-miR-2944b-5p and ast-miR-2944b-5p after KOBAS analysis. (**B**) miRNA-target interaction network of miR-2944b-5p of both the insects showing the shared common targets.

**Figure 5:**
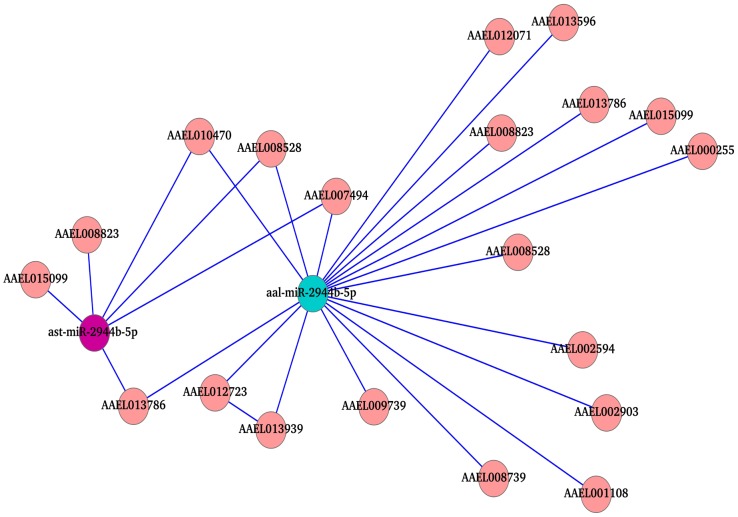
miRNA-targets interaction network of immunity genes for aal-miR-2944b-5p and ast-miR-2944b-5p. Common targets between both the miRNAs are marked as green star.

An ongoing study in our laboratory identified miRNAs from *An. stephensi* and studied their temporal modulation upon *Plasmodium* infection. While analyzing the data, we recognized several common miRNAs between the two vectors namely, *Ae. albopictus* and *An. stephensi*. We now asked this question whether the modulation of these common miRNAs were species/pathogen specific. For this purpose, we studied the differential expression of these miRNAs. Upon analyzing the common miRNAs between the two vectors, 26 miRNA were found to be significant differentially expressed (p value<0.05). We also observed repertoires of miRNAs showing distinct expression pattern upon CHIKV infection in *Aedes* and *Plasmodium* infection in *Anopheles*. Amongst these miRNAs, one miRNA, miR-2944-5p showed up-regulation upon both CHIKV and Plasmodium infections. Similarly, miR-2b showed significant down-regulation in both insects. Targets for these two miRNAs were also predicted, subjected to KOBAS analysis and pathway analysis performed. It was seen that target prediction of aal-miR-2944b-5p revealed 395 targets in *Aedes* and ast-miR-2944b-5p showed 392 targets in *Anopheles*. Four pathways namely, protein processing in endoplasmic reticulum, citrate cycle (TCA cycle),ribosome and ubiquitin mediated proteolysis were found to be common among them (**Fig. 4a**). Network analysis of the targets showed 13 transcripts are shared between the two insects (**Fig. 4b**). In addition to the above mentioned pathways, targets in Immunity cluster were studied in detail in order to understand role of insect immunity during different infections. It was observed that four target genes belonging to Immunity were common to both the miRNAs and only 2 genes interacting with each other in the network (**Figure 5**).

**Figure 6:**
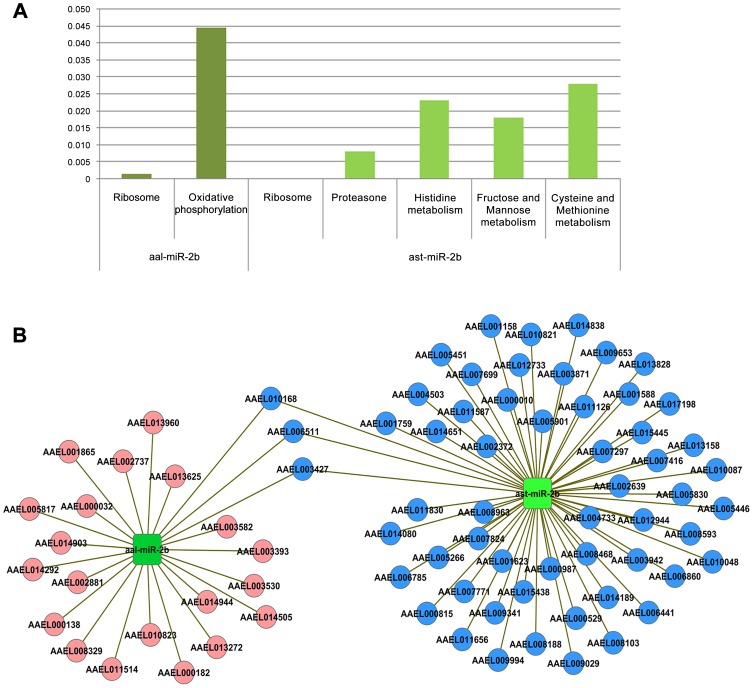
miRNA-targets KOBAS analysis and interaction network of miR-2b of *Anopheles stephensi* and *Aedes aegypti*. (**A**) Graph represents significant pathways of aal-miR-2b and ast-miR-2b after KOBAS analysis. (**B**) miRNA-target interaction network of miR-2b of both the insects showing the shared common targets.

In case of miR-2b, target prediction revealed that aal-miR-2b targeted 226 transcripts, whereas ast-miR-2b had 321 targets. KOBAS analysis of these targets showed two significant pathways for aal-miR-2b and five pathways for ast-miR-2b (**Figure 6a**), of which, ribosome pathway was found to be common. Interaction network of these targets with miRNA showed three common transcripts (AAEL010168, AAEL006511 and AAEL003427) were targeted by both the miRNA homologs (**Figure 6b**). No significant targets of immunity pathways were found for miR-2b.

## Discussion

In this study we have identified miRNAs of a major vector, *Aedes*, using an *Ae. albopictus* cell line and profiled their expression upon CHIKV infection using NextGen sequencing strategies. We identified 90 miRNAs in *Ae. albopictus* of which eight miRNAs common to both uninfected and infected Singh's line were differentially regulated upon CHIKV infection. Through computational biology, we have further predicted targets of the modulated miRNAs and studied the pathways of these targets. Using information of miRNAs that are modulated in *Anopheles* upon *Plasmodium* infection [22], we compared the expression profiles of the common miRNAs in these two distinct vectors upon specific pathogen infection. Even though the data sets derived are from two diverse conditions, i.e., whole insect in case of *Anopheles* and cell line in the case of *Aedes* which is an over-simplified derivation of the whole insect, the findings provide insight to understanding the role of miRNAs as seen in previous reports [33]. Our analysis show different miRNAs that are pathogen/insect specific are up-regulated in the two insects and several of the targets of these miRNAs interact with one another to perform their functions.

Deep sequencing has revealed that distinct repertoire of miRNAs are expressed abundantly in Aedes. It was observed that miR-184 was found to be most abundant in both libraries. Studies have shown that this miRNA multiple roles in female germinal development in *Drosophila* [34]. We also found miRNA-2940-3p to be expressed in abundance in Aedes as reported by Skalsky et al. [33]. A recent study has shown the involvement of this miRNA in *Wolbachia* colonization in Aedes [17]. It is interesting to note that the expression of miR-2940 was down-regulated upon CHIKV infection. Detailed studies are needed to understand the interplay between *Wolbachia* and other viruses in Aedes and the role this miRNA might play in their growth and development.

Upon CHIKV infection, we showed that distinct miRNAs, namely, miR-100, miR-305-3p, miR-283 and miR-927-5p were up-regulated in *Aedes*. Likewise, we also showed that miR-1000, miR-2b, miR-2c and miR-190-5p were down-regulated upon CHIKV infection.We used computational biology to predict the targets of these miRNAs. Using KOBAS, we identified that several metabolic and signaling pathways were altered upon CHIKV infection, with protein processing in endoplasmic reticulum being the common pathway in three of the four up-regulated miRNAs analyzed. Viral replication is active in the first 24 hours of infection and most of the host transcription machinery is hijacked by the virus which could be the reason for these pathways being affected upon infection. Another pathway that was significantly altered was part of the immunity pathway showcasing the involvement of insect immunity upon pathogen infection and the role microRNAs may be playing in regulation of insect immune system.

We further proceeded to evaluate the interactions of the predicted transcripts in these pathways with the miRNAs. We observed that several of the transcripts were common among the miRNAs. Of interest were three transcripts that were common among three of the four up-regulated miRNAs. It was seen that these three transcripts encoded for a protein tyrosine phosphatase (SHP2), ERK1/2 and an ubiquitin fusion degradation protein. Similarly, one transcript, a 40S ribosomal protein S16, was common is three of the four miRNAs down-regulated. Each of these targets is important in different aspects of host response to virus pathogenesis as is evidenced by several reports. It is known that viruses activate extracellular signal regulated kinases(ERKs) during infection [35], [36]. A recent study has highlighted the importance of this kinase in antiviral defense in insects during arbovirus infection [37].Similarly, it is known virus entry is regulated by SHP2 and it upstream modulators [38].Similarly, ribosomal proteins (AAE003427, AAEL010168) were common targetsin miRNAs down-regulated during CHIKV infection.

Further, we hypothesized that comparison of different vectors upon different pathogen infections will yield pathogen-vector specific modulators. For this purpose, we compared the miRNA population of Aedes infected with CHIKV and Anopheles infected with *Plasmodium*. We identified 76 common miRNAs in the two insect datasets, of which one miRNA, miR-2944-5p, was significantly up-regulated and one miRNA, miR-2b, that was significantly down-regulated. We argued that predicting the targets of these miRNA homologs in the two insects will result in distinct targets and provide information as to the host cell regulators to specific pathogens. As seen in the other up-regulated miRNAs, signaling and metabolic pathways were most altered in miR-2944-5p upon *Plasmodium* or CHIKV infection. In addition, immunity pathway was also studied in detail and revealed interesting information. Pathway analysis revealed four genes, AAEL007494, AAEL010470 (both coding for a calcium binding protein), AAEL008528 (a tyrosine protein phosphatase non-receptor type 11 (SHP2), AAEL013786 (growth factor receptor binding protein 2). Literature search for these proteins revealed that a growth factor receptor binding protein 2 (GRB 2) binds to NS5A of HCV (Hepatitis C virus) in a homology 3 domain/ligand-dependent manner and perturbs mitogenic signaling [39]. Another study has implicated GRB2 (Growth factor receptor-bound protein 2) in murine leukemia virus entry through its interaction with ectotropic MLV receptor [40]. A more recent study has shown that Grb2 controls phosphorylation of FGFR2 (Fibroblast growth factor receptor 2) by inhibiting receptor kinase and SHP2 phosphatase activity [38]. It is significant to note that SHP2 phosphatase is another target of miR-2944-5p as well as the three miRNAs up-regulated that was discussed earlier in this study.

Our present work has identified known and novel miRNAs in *Ae. albopictus*. We have analyzed the distribution and abundance of these miRNAs and evaluated their modulation upon CHIKV infection. Target prediction and pathway analysis of these miRNAs were also performed throwing light to the possible role of these miRNAs. Further comparison of some of these Aedes miRNAs with data generated from An. stephensi miRNAs regulate upon *Plasmodium* infection has revealed interesting information on microRNAs of these two important vectors, upon pathogen infection. While *An. stephensi* is an important urban vector of South East Asia, *Ae.albopictus* is gaining notoriety for the spread of chikungunya and more recently dengue. Analysis of the expression of miRNAs and their putative targets in our study have provided important action points for studying the basis of pathogenesis in these vectors.

## Supporting Information

Figure S1 Pictorial representation of the workflow followed for identification and analysis of non-coding RNAs and targets in this study. (**A**) The figure represents the workflow for identifying known miRNA, novel miRNA and their targets using 3′UTR sequences. (**B**) The figure represents workflow for the identification of targets of significantly upregulated miRNAs and downregulated miRNAs and KOBAS pathway analysis of the targets for the identification of significant pathways.
(TIF)

Figure S2 The figure represents the structures of novel miRNAs identified in this study.
(TIF)

Table S1 List of common miRNAs between SL and iSL libraries identified in this study. miRNA sequences and TPM values are written along with the miRNAs.
(DOCX)
