# Supplementary figures and images for: Next Generation Sequencing Reveals Regulation of Distinct Aedes microRNAs during Chikungunya Virus Development
Source: PLoS Negl Trop Dis. 2014 Jan 9;8(1):e2616. doi: 10.1371/journal.pntd.0002616 (PMC3888459; doi:10.1371/journal.pntd.0002616)

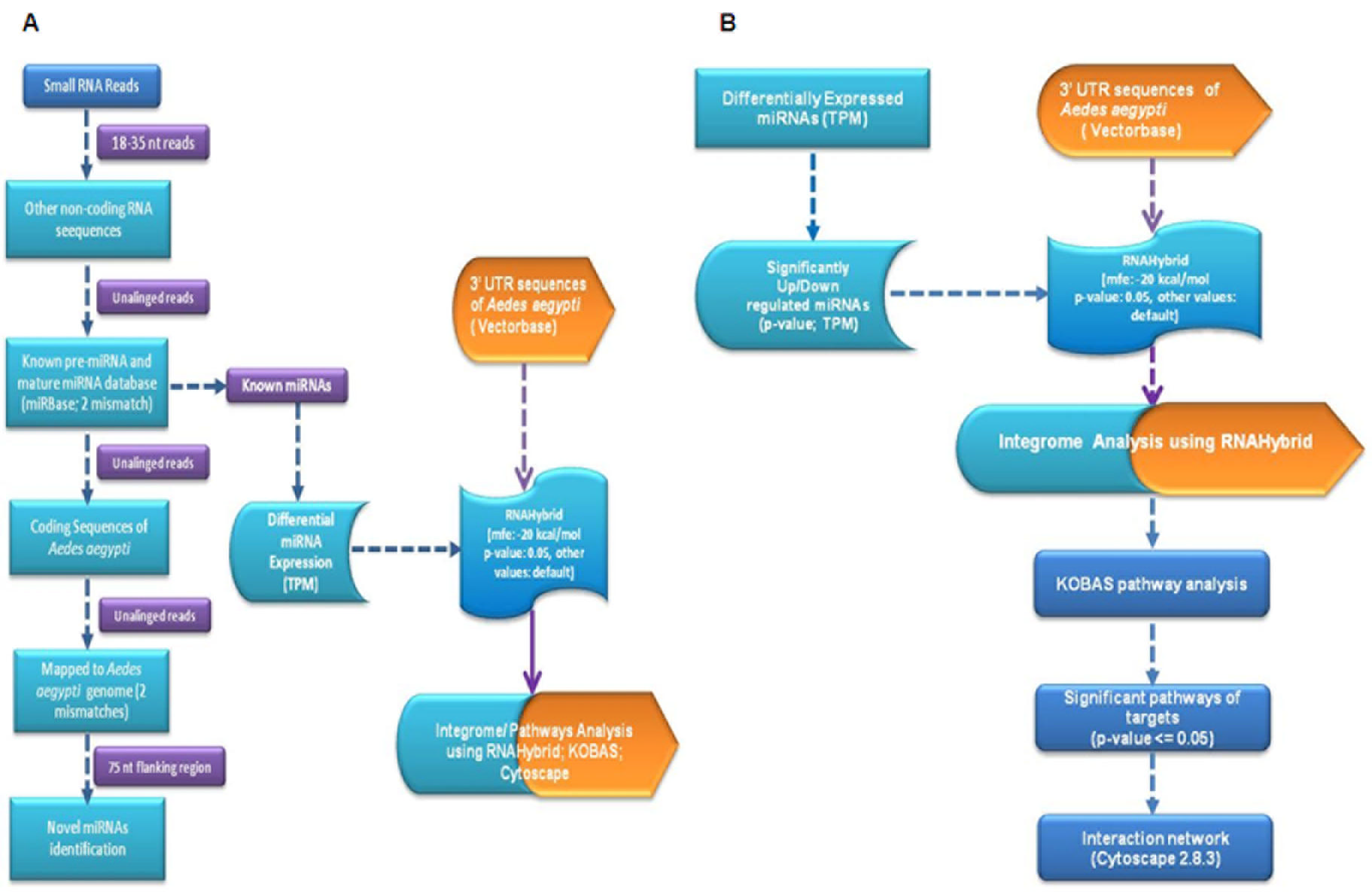

Supplement: Figure S1 — Pictorial representation of the workflow followed for identification and analysis of non-coding RNAs and targets in this study. (A) The figure represents the workflow for identifying known miRNA, novel miRNA and their targets using 3′UTR sequences. (B) The figure represents workflow for the identification of targets of significantly upregulated miRNAs and downregulated miRNAs and KOBAS pathway analysis of the targets for the identification of significant pathways. (TIF) [file pntd.0002616.s001.tif]

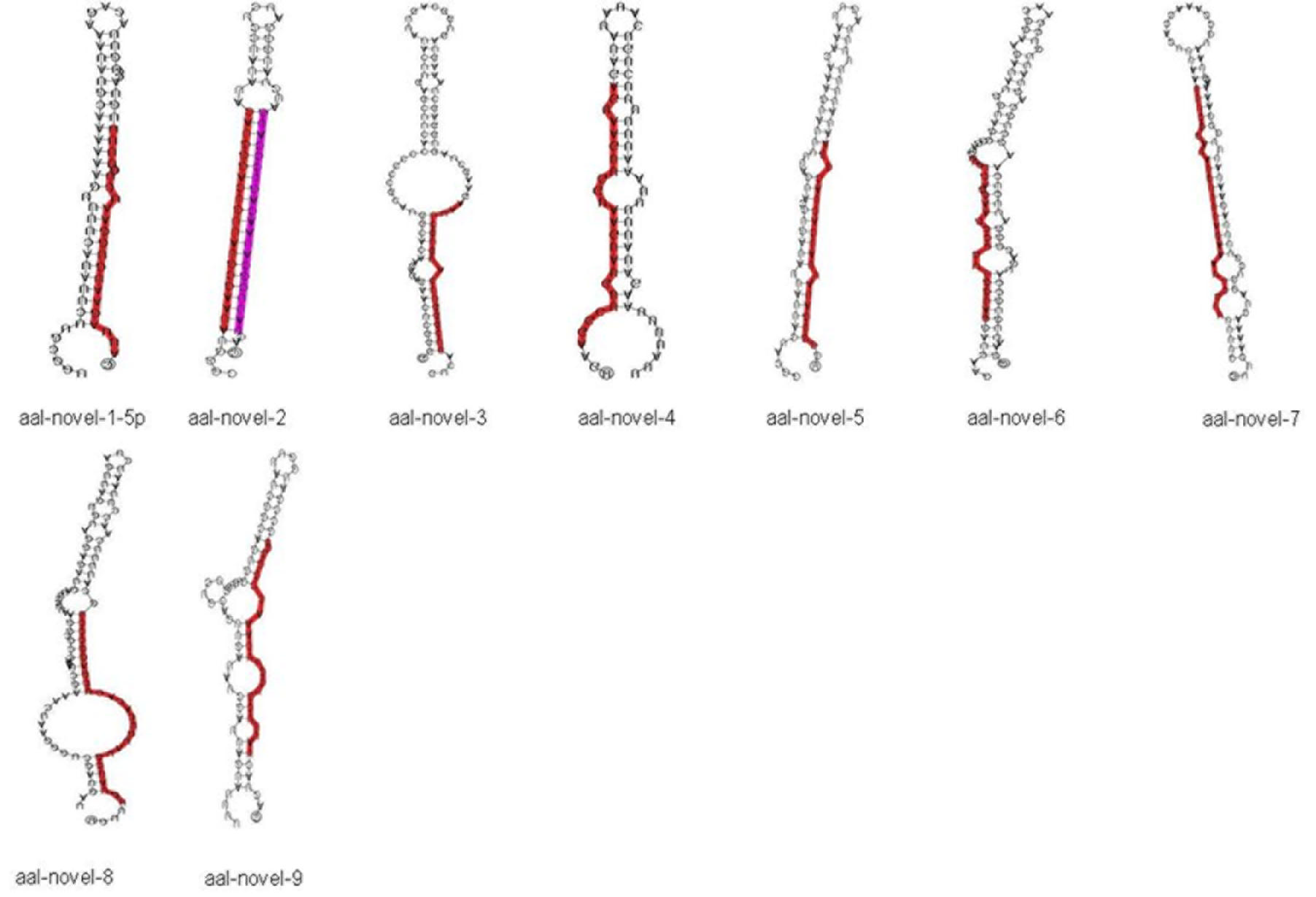

Supplement: Figure S2 — The figure represents the structures of novel miRNAs identified in this study. (TIF) [file pntd.0002616.s002.tif]
