# Supplementary material for: Next Generation Sequencing Reveals Regulation of Distinct Aedes microRNAs during Chikungunya Virus Development
Source: PLoS Negl Trop Dis. 2014 Jan 9;8(1):e2616. doi: 10.1371/journal.pntd.0002616 (PMC3888459; doi:10.1371/journal.pntd.0002616)
Supplement: Table S1 — List of common miRNAs between SL and iSL libraries identified in this study. miRNA sequences and TPM values are written along with the miRNAs. (DOCX) [file pntd.0002616.s003.docx]

**Table S1.** Common miRNAs between SL and iSL.

| **S. No.** | **Length** | **Sequence** | **miRNA** | **SL** | **iSL** |
| --- | --- | --- | --- | --- | --- |
| 1 | 23 | TGAGATCATTTTGAAAGCTGATT | bantam-3p | 11771.15 | 7066.99 |
| 2 | 23 | CCGGTTTTCATTTTCGATCTGAC | bantam-5p | 17.81 | 23.17 |
| 3 | 21 | TGAGGTAGTTGGTTGTATAGT | let-7 | 2786.44 | 1912.68 |
| 4 | 22 | AACCCGTAGATCCGAACTTGTG | miR-100 | 4167.34 | 5236.15 |
| 5 | 21 | ATATTGTCCTGTCACAGCAGT | miR-1000 | 3.82 | 0.87 |
| 6 | 22 | TACCCTGTAGAACCGAATTTGT | miR-10-5p | 4.51 | 2.6 |
| 7 | 22 | CATCACAGTCAGAGTTCTTGCT | miR-11-3p | 6812.69 | 4749.86 |
| 8 | 21 | ACAAGTTTTGATCTCCGGTAT | miR-125-3p | 38.86 | 64.3 |
| 9 | 22 | TCCCTGAGACCCTAACTTGTGA | miR-125-5p | 270.61 | 327.58 |
| 10 | 23 | TGAGTATTACATCAGGTACTGGT | miR-12-5p | 383.82 | 295.76 |
| 11 | 22 | TTGGTCCCCTTCAACCAGCTGT | miR-133-3p | 1.5 | 0.65 |
| 12 | 23 | TATCACAGCCATTTTGACGAGTT | miR-13-3p | 539.59 | 332.78 |
| 13 | 21 | TCGTAAAAATGGTTGTGCTGT | miR-13-5p | 20.47 | 16.02 |
| 14 | 20 | TCAGTCTTTTTCTCTCTCCT | miR-14 | 7725.12 | 5398.97 |
| 15 | 22 | TGGACGGAGAACTGATAAGGGC | miR-184-3p | 81027.58 | 83651.55 |
| 16 | 22 | TGAAATCTTTGATTAGGTCTGG | miR-1890 | 24.52 | 16.45 |
| 17 | 22 | CCCAGGAATCAAACATATTATT | miR-190-3p | 0.46 | 0.22 |
| 18 | 24 | AGATATGTTTGATATTCTTGGTTG | miR-190-5p | 219.38 | 78.59 |
| 19 | 21 | CTTGTGCGTGTGACAACGGCT | miR-210-3p | 0.93 | 0.87 |
| 20 | 19 | CTGCTGCCCAAGTGCTTAT | miR-252-3p | 12.26 | 5.63 |
| 21 | 21 | CTAAGTACTAGTGCCGCAGGA | miR-252-5p | 1719.74 | 1768.7 |
| 22 | 21 | AATGGCACTGGAAGAATTCAC | miR-263a-5p | 8.21 | 4.55 |
| 23 | 22 | TCAGGTACCTGAAGTAGCGCGC | miR-275-3p | 2392.56 | 3182.31 |
| 24 | 22 | TAGGAACTTCATACCGTGCTCT | miR-276-3p | 4000.47 | 4445.45 |
| 25 | 22 | TGGTAACTCCACCACCGTTGGC | miR-2765 | 191.16 | 161.95 |
| 26 | 22 | AGCGAGGTATAGAGTTCCTACG | miR-276-5p | 3.35 | 3.25 |
| 27 | 22 | TAAATGCACTATCTGGTACGAC | miR-277-3p | 2673.22 | 2318.64 |
| 28 | 18 | ATCCGGCTCGAAGGACCA | miR-2779 | 13.18 | 14.72 |
| 29 | 21 | TCGGTGGGACTTTCGTCCGTT | miR-278-3p | 60.83 | 36.37 |
| 30 | 20 | TGACTAGATCCACACTCATT | miR-279 | 338.95 | 281.03 |
| 31 | 22 | TGTCATGGAATTGCTCTCTTTA | miR-281-3p | 20.82 | 15.59 |
| 32 | 22 | AAGAGAGCTGTCCGTCGACAGT | miR-281-5p | 4829.63 | 5092.82 |
| 33 | 21 | CAATATCAGCTGGTAATTCTG | miR-283 | 1.73 | 5.85 |
| 34 | 22 | TAGCACCATTCGAAATCAGTAC | miR-285 | 0.46 | 0.22 |
| 35 | 22 | TGACTAGACCGAACACTCGCGT | miR-286a | 5.32 | 6.06 |
| 36 | 22 | GTCGACAGGGAGATAAATCACT | miR-2940-3p | 32669.5 | 16188.68 |
| 37 | 19 | TATCACAGCAGTAGTTACC | miR-2944b-3p | 4.39 | 4.76 |
| 38 | 23 | GAAGGAACTCCCGGTGTGATATT | miR-2944b-5p | 13.65 | 15.37 |
| 39 | 21 | TGACTAGAGGCAGACTCGTTT | miR-2945-3p | 279.05 | 166.07 |
| 40 | 18 | AGCTCAGCACGCAGGGGC | miR-2951-5p | 0.35 | 0.43 |
| 41 | 21 | TATCACAGCCAGCTTTGAAGA | miR-2a-3p | 1043.34 | 911.52 |
| 42 | 24 | TATCACAGCCAGCTTTGATGAGCT | miR-2b | 925.73 | 348.15 |
| 43 | 18 | TATCACAGCCAGCTTTGA | miR-2c-3p | 11.56 | 2.38 |
| 44 | 22 | CGGCACATGTTGGAGTACACTT | miR-305-3p | 111.25 | 319.79 |
| 45 | 24 | ATTGTACTTCATCAGGTGCTCTGG | miR-305-5p | 689.82 | 509.24 |
| 46 | 22 | GAGAGCACCTCGGTATCTAAGC | miR-306-3p | 0.35 | 0.87 |
| 47 | 21 | TCACAACCTCCTTGAGTGAGC | miR-307 | 1.62 | 2.17 |
| 48 | 19 | AATCACAGGAGTATACTGT | miR-308-3p | 23.71 | 21.22 |
| 49 | 21 | TCACTGGGCAAAGTTTGTCGC | miR-309a | 43.37 | 32.69 |
| 50 | 21 | TGGCAAGATGTTGGCATAGCT | miR-31 | 0.58 | 0.22 |
| 51 | 23 | TTTTGATTGTTGCTCAGAAAGCC | miR-315 | 1.04 | 0.22 |
| 52 | 21 | GTGCATTGTAGTTGCATTGCA | miR-33-5p | 35.97 | 15.81 |
| 53 | 23 | TGGCAGTGTGGTTAGCTGGTTGT | miR-34-5p | 89.05 | 59.76 |
| 54 | 22 | TTTGTTCGTTTGGCTCGAGTTA | miR-375 | 3.58 | 1.73 |
| 55 | 20 | TCTCACTACCTTGTCTTTCA | miR-71-3p | 745.9 | 596.28 |
| 56 | 22 | AGAAAGACATGGGTAGTGAGAT | miR-71-5p | 20.47 | 20.14 |
| 57 | 23 | TGGAAGACTAGTGATTTTGTTGT | miR-7-5p | 15.03 | 6.71 |
| 58 | 21 | ATAAAGCTAGATTACCAAAGC | miR-79-3p | 16.07 | 14.51 |
| 59 | 22 | CTTTGGCGCTTTAGCTGTATGA | miR-79-5p | 1.04 | 1.52 |
| 60 | 23 | TAATACTGTCAGGTAAAGATGTC | miR-8-3p | 2808.76 | 2397.23 |
| 61 | 22 | CATCTTACCGGGCAGCATTAGA | miR-8-5p | 281.01 | 457.28 |
| 62 | 22 | TTTAGAATTCCTACGCTTTACC | miR-927a | 0.46 | 1.73 |
| 63 | 21 | AAATTGACTCTAGTAGGGAGT | miR-929-5p | 0.46 | 0.87 |
| 64 | 20 | TATTGCACTTGTCCCGGCCT | miR-92a-3p | 14.46 | 22.3 |
| 65 | 22 | AATTGCACTTGTCCCGGCCTGC | miR-92b-3p | 128.48 | 95.48 |
| 66 | 22 | TGAAACCGTCCAAAACTGAGGC | miR-957 | 1.73 | 1.3 |
| 67 | 21 | TAAGCGTATAGCTTTTCCCAT | miR-965 | 1.39 | 0.87 |
| 68 | 21 | TCATAAGACACACGCGGCTAT | miR-970 | 558.56 | 514.44 |
| 69 | 20 | TAGCTGCCTAGTGAAGGGCT | miR-980-3p | 333.86 | 282.55 |
| 70 | 22 | CCCCTTGTTGCAAACCTCACGC | miR-988-3p | 58.17 | 71.88 |
| 71 | 21 | TGTGATGTGACGTAGTGGTAC | miR-989 | 0.46 | 0.22 |
| 72 | 20 | TGACTAGATTACATGCTCGT | miR-996 | 263.9 | 205.25 |
| 73 | 21 | TAGCACCATGAGATTCAGCTC | miR-998-3p | 47.88 | 58.89 |
| 74 | 22 | TGTTAACTGTAAGACTGTGTCT | miR-999 | 349.13 | 249.21 |
| 75 | 21 | TCTTTGGTTATCTAGCTGTAT | miR-9a | 0.23 | 0.87 |
| 76 | 22 | TAAAGCTTTAGTACCAGAGGTC | miR-9c-3p | 100.73 | 119.08 |
| 77 | 22 | TCTTTGGTATTCTAGCTGTAGA | miR-9c-5p | 133.11 | 78.16 |
